# Hippocampal neuronal hypoexcitability contributes to PTSD-like phenotypes in the experimental autoimmune encephalomyelitis model

**DOI:** 10.3389/fpsyt.2026.1683599

**Published:** 2026-05-20

**Authors:** Xinghua Zhong, Han Zhang, Jieying Xie, Yang Gao, Honghao Wang, Yu Peng, Feng Yi, Jinyu Chen

**Affiliations:** 1 Key Laboratory of Mental Health of the Ministry of Education, Guangdong-Hong Kong-Macao Greater Bay Area Center for Brain Science and Brain-Inspired Intelligence, Guangdong-Hong Kong Joint Laboratory for Psychiatric Disorders, Guangdong Province Key Laboratory of Psychiatric Disorders, Guangdong Basic Research Center of Excellence for Integrated Traditional and Western Medicine for Qingzhi Diseases, Department of Neurobiology, School of Basic Medical Sciences, Southern Medical University, Guangzhou, China; 2 Department of Neurology, Guangzhou First People’s Hospital, School of Medicine, Southern China University of Technology, Guangzhou, China; 3 Department of Neurology, Nanfang Hospital, Southern Medical University, Guangzhou, China

**Keywords:** experimental autoimmune encephalomyelitis, fear−extinction, hippocampal CA1, neuronal hypoexcitability, posttraumatic stress disorder

## Abstract

**Introduction:**

Patients with multiple sclerosis (MS) frequently exhibit hippocampal atrophy and post-traumatic stress disorder (PTSD)–like symptoms, yet the neural mechanisms linking these clinical features remain unclear.

**Methods:**

Using the experimental autoimmune encephalomyelitis (EAE) mouse model of MS, we employed morphometric analysis, whole-cell recordings, chemogenetic manipulation, and single-nucleus RNA sequencing to investigate the structural, functional, and transcriptional changes in the hippocampus.

**Results:**

EAE animals recapitulated two core PTSD-relevant phenotypes: impaired fear extinction and contextual fear generalization. Morphometric analysis revealed somatic atrophy of dorsal CA1 pyramidal neurons, and whole-cell recordings demonstrated a marked reduction in firing gain, indicating hippocampal hypoexcitability. Chemogenetic reactivation of CA1 pyramidal neurons ameliorated extinction deficits in EAE mice. Single-nucleus RNA sequencing further revealed a robust neuroimmune-like transcriptional shift in CA1 excitatory neurons, characterized by enrichment of immune/interferon-associated programs (including antigen processing and presentation and MHC-related pathways) together with apoptosis-related gene signatures.

**Discussion:**

Collectively, these data link neuroinflammation-associated neuronal reprogramming to CA1 structural and functional impairment and suggest that restoring hippocampal excitability may represent a therapeutic avenue for MS patients with comorbid PTSD-like symptoms.

## Introduction

Multiple sclerosis (MS) is a chronic, immune-mediated demyelinating disorder of the central nervous system. Beyond motor and sensory deficits, 30–70% of patients develop clinically relevant neuro-psychiatric symptoms, including executive-function deficits, anxiety, and depression (1). Large epidemiological and clinical cohorts show a substantially higher risk of post-traumatic stress disorder (PTSD) among people with MS, with point prevalence of 4–6% and self-reported PTSD-like symptoms in up to 25–50% of patients, although full DSM-5 criteria are not met (2–4). Patients with relapsing MS and comorbid PTSD exhibit higher relapse rates, increased brain magnetic resonance imaging (MRI) lesion burden, and faster disability progression (5). It also erodes treatment adherence, worsens mood, lowers quality of life, and increases healthcare costs; targeted psychological treatments such as eye-movement desensitization and reprocessing (EMDR) can partly reverse these effects (6). The biological mechanisms that make MS patients susceptible to PTSD, however, remain poorly understood.

Mounting evidence highlights the hippocampus, particularly its dorsal subdivision (dHPC), as a shared pathological hub in MS and PTSD. Neuro-imaging and neuropathological studies reveal early demyelination, microglial activation, and selective hippocampal atrophy in MS, with the extent of volume loss closely tracking memory impairment (7–9). Parallel work in PTSD demonstrates reduced hippocampal volume and blunted functional responses that correlate with symptom load and treatment outcome (10).

Experimental autoimmune encephalomyelitis (EAE), the canonical murine model of MS, recapitulates these hippocampal abnormalities. The dHPC shows robust microglial activation and synaptic pathology (11), diminished intrinsic excitability of CA1 pyramidal neurons driven by enhanced tonic GABAergic inhibition (12), and impaired long-term potentiation (13). Rodent PTSD models likewise display stress-induced hippocampal inflammation that contributes to deficits in fear-extinction learning, a process critically dependent on dHPC integrity (14).

Given the converging evidence that hippocampal dysfunction, especially dorsal CA1 hypo-excitability, emerges in both multiple sclerosis (MS) and post-traumatic stress disorder (PTSD), yet mechanistic links between the two conditions remain undefined, we hypothesize that autoimmune demyelination precipitates a hypo-excitable state in dorsal CA1 pyramidal neurons that impairs fear-extinction function and drives PTSD-like symptoms in MS.

To test this hypothesis, we will first assess fear-extinction deficits and their generalization in experimental autoimmune encephalomyelitis (EAE) mice and, by combining immunofluorescence histology with whole-cell patch-clamp recordings, systematically map functional abnormalities in the affected brain regions and neuron subtypes. We will then transiently boost dorsal CA1 excitability with hM3Dq chemogenetics to determine whether restoring neuronal firing ameliorates the extinction deficit, thereby establishing causality. Finally, single-nucleus RNA sequencing will define the transcriptional program that may drive these impairments. This multi-scale, integrative strategy will clarify the mechanisms underpinning MS-PTSD comorbidity and identify neuro-immune targets for therapeutic intervention.

## Materials and methods

### Induction and scoring of experimental auto-immune encephalomyelitis

Female C57BL/6J mice (7 weeks old, 18–20g) were housed 4–5 per cage under a 12 h light/dark cycle (lights on 07:00, 22 ± 1 °C, 50 ± 5% humidity) with food and water ad libitum. All procedures were conducted in accordance with the Guidelines for the Care and Use of Laboratory Animals of Southern Medical University and were approved by the Ethics Committee of Southern Medical University. The study is reported in accordance with the ARRIVE guidelines (https://arriveguidelines.org).

Experimental autoimmune encephalomyelitis (EAE) was induced based on a previously established method, with minor modifications (15). MOG_3_^5^–^55^ peptide (MEVGWYRSPFSRVVHLYRNGK; GL Biochem, Shanghai, China) was emulsified at a final concentration of 1 mg/mL in Complete Freund’s adjuvant containing 4 mg/mL Mycobacterium tuberculosis H37Ra (Chondrex Inc., Cat #7001) at 4 °C. On day 0, mice received 150 µL of this emulsion by subcutaneous injection at two dorsal sites, immediately followed by intraperitoneal administration of 200 µL pertussis toxin working solution (1 µg/mL; List Biologicals, USA). A second dose of pertussis toxin (200 µL) was given intraperitoneally on day 3. Clinical signs and body weight were monitored every other day by a blinded investigator using a 0–5 scoring scale (0.0: Normal;0.5: Tail−tip droop;1.0: Tail limp or ataxic gait;1.5: Tail limp + ataxia;2.0: Partial paralysis of both hindlimbs or full paralysis of one hindlimb;2.5: Full paralysis of one hindlimb + partial paralysis of the other;3.0: Full paralysis of both hindlimbs;3.5: Paralysis of both hindlimbs + weakness in one forelimb;4.0: Paralysis of all four limbs;5.0: Death).

### Open field test

Spontaneous locomotor activity was quantified in a conventional open-field paradigm. Eight identical arenas (inner dimensions: 40 cm × 40 cm × 30 cm; Omnitech Electronics, USA) were used, each fitted with an array of infrared sensors that continuously detected horizontal movement. At the start of the session, a single mouse was gently placed at the center of an arena and allowed to explore freely for 10 min under standard room illumination (≈ 200 lux). Between trials, arenas were cleaned with 70% ethanol to remove olfactory cues.

For each animal, total distance travelled across the full 10-min session served as the primary locomotor read-out. Anxiety-related behaviors were inferred from dwell time in the center zone of the open field during 10 min. The center zone was defined as a 20 cm × 20 cm square positioned equidistant from the arena walls. Time spent in this zone was extracted from the activity file and expressed both as absolute duration (seconds) and as a percentage of the 10-min interval.

### Thermal nociception

Thermal nociception was measured on a constant-temperature hot plate (BIO-CHP, Bioseb, France) maintained at 53 ± 0.2 °C. Mice were habituated to the test room (22 ± 2 °C) for 30 min before testing. Each mouse was placed in an acrylic cylinder on the plate, and the latency to the first nocifensive response (hind-paw lick) was recorded as pain latency. A 30-s cut-off was used to prevent tissue injury; animals not responding were removed and assigned 30 s. Three trials per mouse were performed ≥15 min apart, and the mean pain latency was used for analysis. Experimenters were blinded to group, and test order was randomized.

### Contextual fear conditioning, retrieval, and generalization

Contextual fear conditioning was performed according to a previously described protocol (16) with minor modifications. We employed a relatively strong foot‐shock stimulation (1 mA) during fear memory training to test the susceptibility to stress induced PTSD like phenotype of EAE and control mice. On the training day, mice were placed in a Plexiglas chamber (32 × 26 × 25 cm; stainless−steel grid floor, Med Associates, USA) that had been wiped with 70 % ethanol. After a 180 s baseline exploration period, four foot−shocks (1 mA, 2 s) were delivered at 60 s intervals. Mice remained in the chamber for 120 s after the final shock before being returned to their home cages.

Twenty−four hours later, contextual memory retrieval was evaluated by re−exposing each mouse to the same chamber for 30 min without shock. Freezing, defined as complete immobility except for respiration for ≥ 1 s, was scored automatically with ANY−maze software (Stoelting Co, USA) whose algorithm has been validated against manual scoring. Chambers were cleaned with 1 % acetic acid between animals. To assess fear−memory retention, mice were returned to the original conditioning chamber (Context A) on post−training days 8 and 14; fear generalization was probed on day 9 by placing the animals in a novel chamber (Context B) with a white−paperboard floor and patterned wall inserts. Freezing in Context B was quantified using the same apparatus, session length, and scoring parameters as in Context A.

### Immunofluorescence staining

Mice were deeply anaesthetized with Avertin (100 mg kg^-^¹, i.p.) and transcardially perfused with ice−cold phosphate−buffered saline (PBS), followed by 4 % paraformaldehyde (PFA) in PBS. Brains were post−fixed in 4 % PFA at 4 °C for 8 h, cryoprotected in 30 % sucrose, and coronally sectioned at 40 µm on a Leica CM1950 cryostat. Free−floating sections were blocked for 2 h at room temperature in 10 % normal donkey serum containing 0.3 % Triton X−100 and then incubated overnight at 4 °C with one of the following primary antibodies: rabbit anti-IBA1 (1:1000, Wako #019−19741), mouse anti−NeuN (1:500, Millipore MAB377), or Guinea pig anti−c−Fos (1:5000, Synaptic Systems #226 308). After PBS washes, sections were incubated for 2 h at room temperature with species−appropriate donkey secondary antibodies conjugated to Alexa Fluor 488 or Alexa Fluor 594 (1:500, Jackson ImmunoResearch), counterstained with DAPI (0.1 µg mL^-^¹), and mounted in ProLong™ Glass. Confocal images were acquired on a Nikon A1 microscope with identical laser−power and detector settings across all groups. Fluorescence intensity and cell counts were quantified in FIJI by investigators blinded to the experimental conditions.

### Whole-cell recordings using brain slices

Deeply anaesthetized mice (isoflurane) were transcardially perfused with ice-cold, oxygenated (95% O_2_/5% CO_2_) slicing solution. Brains were rapidly removed and immediately placed in the same ice-cold, oxygenated solution. Coronal slices (300 µm) were cut on a VT1200S vibratome (Leica Microsystems, Wetzlar, Germany) and incubated at 34 °C for 30 min, then transferred to recording artificial cerebrospinal fluid (ACSF; in mM: 125 NaCl, 2.5 KCl, 1.25 NaH_2_PO_4_, 25 NaHCO_3_, 20 glucose, 2 CaCl_2_, 1 MgCl_2_; pH 7.35; 295–300 mOsm) and held at room temperature until use.

Whole-cell patch-clamp recordings were obtained from CA1 pyramidal neurons visualized with inferred optics. Patch pipettes (4–6 MΩ) were filled with an internal solution containing (in mM): 125 K-Gluconate, 10 HEPES, 4 KCl, 4 MgATP, 0.3 NaGTP, 10 Na-Phosphocreatine, and 0.2 EGTA (pH 7.25; 295 mOsm). Signals were amplified with a HEKA EPC10 USB amplifier, lowpass filtered at 8 kHz, digitised at 20 kHz, and acquired using PatchMaster software on a PC for offline analysis with Axograph X (AxoGraph Scientific, Sydney, Australia).

### Chemogenetic manipulation of dCA1 neuronal activity

We stereotaxically delivered a 1:1 mixture of AAV2/9-DIO-hM3Dq-GFP (or AAV2/9-DIO-GFP for controls; 5 × 10¹² vg mL^-^¹, Heyuan Bio) and AAV2/9-CaMKIIα-Cre (2 × 10¹² vg mL^-^¹, Brain VTA) bilaterally into the dorsal hippocampus (AP -1.8 mm, ML ± 1.6 mm, DV −1.3 mm relative to bregma). Injections were made at 50 nL min^-^¹ (300 nL per site), and the needle was left in place for 10 min to minimize reflux. Animals recovered for at least 3 weeks before further procedures.

For chemogenetic activation, deschloroclozapine (DCZ; MedChemExpress, HY-42110) was freshly prepared by diluting the stock solution in physiological saline to a final concentration of 0.03 mg/mL and administered intraperitoneally at a dose of 0.3 mg/kg 1 h before behavioral testing. All behavioral and electrophysiological assessments were conducted by investigators blinded to treatment allocation.

### Single-nucleus RNA sequencing

Hippocampi from peak EAE mice (MOG_3_^5^-^55^/CFA, clinical score ≈ 2) and age-matched adjuvant controls were perfused with ice-cold PBS, minced, and digested for 30 min at 37 °C in collagenase I/II plus DNase I according to the manufacturer’s recommendations. Cell suspensions were filtered (70 µm, then 30 µm), red cells lysed, and nuclei resuspended in DPBS/0.04% BSA. Libraries were prepared with the LeadOmics 3′ scRNA kit (cat. 10401-001419) and sequenced on an MGI DNBSEQ-T7. Read1 contained a 20-bp cell barcode and 10-bp UMI; Read2 comprised 100 bp of transcript. Raw data were processed with Cell Sketcher v1.0: barcode correction, STAR v2.7.11b alignment to mm10, UMI deduplication and gene counting. In Seurat v5.1.0, the top 2, 000 variable genes per sample were integrated by CCA (dims = 30), followed by PCA, UMAP, and Louvain clustering (resolution = 0.5). Differential expression was assessed by Wilcoxon test (FDR < 0.05), with robust DE genes defined as Up32 (log_2_FC > 1) and Down32 (log_2_FC < −1); cell types were annotated with SingleR v2.8.0, followed by GO/KEGG enrichment using clusterProfiler (FDR ≤ 0.05). Pathway enrichment analysis was performed using the Kyoto Encyclopedia of Genes and Genomes (KEGG) database (Kanehisa Laboratories; https://www.kegg.jp) (17).

### Data and statistical analyses

Electrophysiological recordings obtained from mouse brain slices were processed and analyzed with Axograph software (Axograph Scientific, Sydney, Australia). All statistical analyses were conducted in GraphPad Prism 10 (GraphPad Software) using unpaired two-tailed t-tests (Welch-corrected for unequal variances), two-way ANOVA, or unpaired Student’s t-tests as experimental designs dictated. Normality and homogeneity of variance were verified by Shapiro-Wilk and Levene’s tests respectively. For significant ANOVA main effects/interactions (*p < 0.05), Bonferroni-adjusted *post hoc* analyses were performed. Data are presented as mean ± SEM throughout.

## Results

### EAE mice exhibit PTSD-like phenotypes

**Figure 1:**
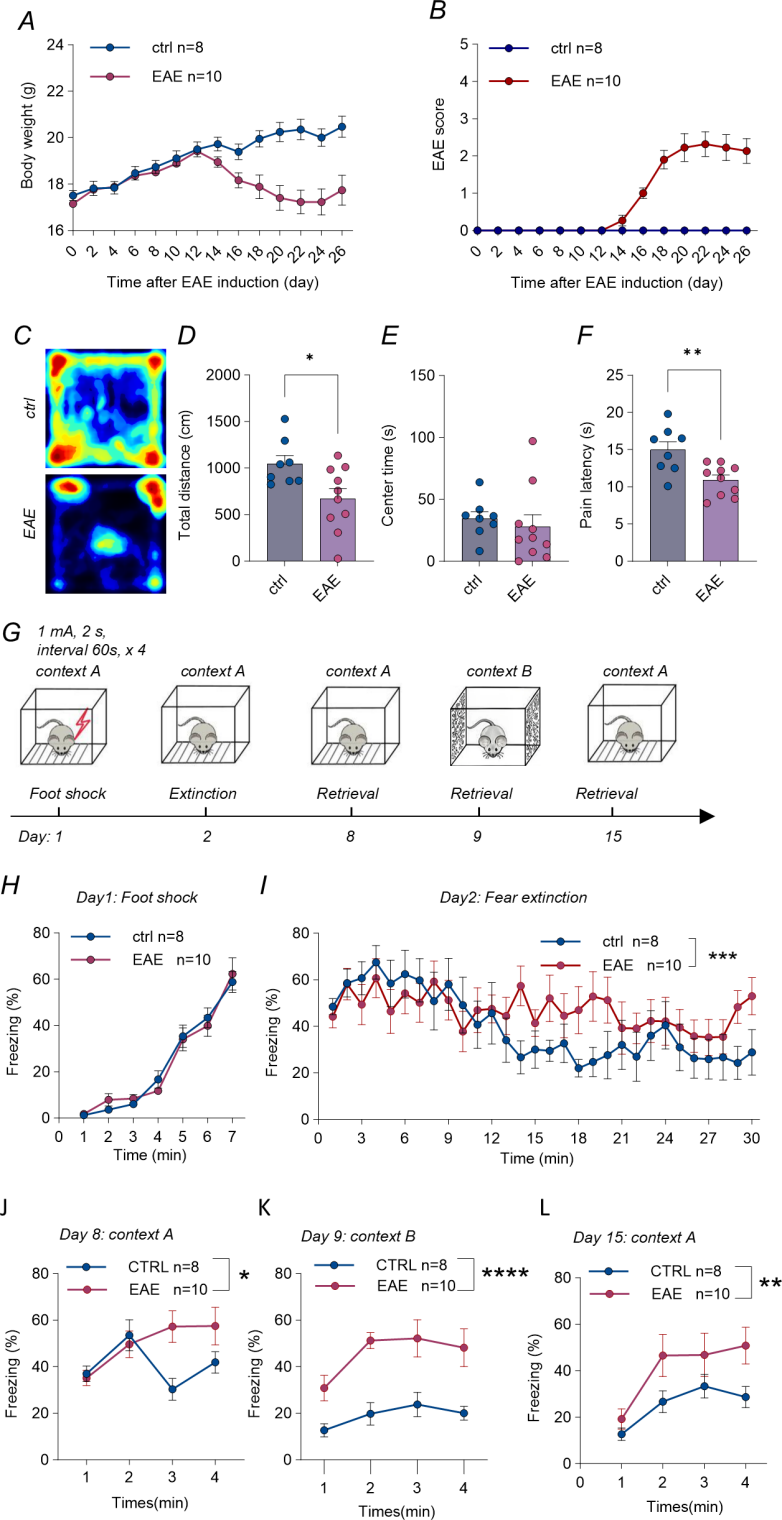
EAE elicits PTSD-like behavioral phenotypes. **(A)** Body weight changes after EAE induction in control and groups. EAE mice show significant weight loss after disease onset. (Ctrl, n=8; EAE, n=10). **(B)** Clinical EAE scores over time in ctrl and EAE groups, demonstrating disease progression in EAE mice. (Ctrl, n=8; EAE, n=10). **(C)** Representative movement tracks in the open field test for ctrl and EAE mice. **(D)** Total distance traveled in the open field is significantly reduced in EAE mice compared to controls (ctrl, n=8 vs EAE, n=10; 1048 ± 87.82 vs 673.2 ± 109.9, unpaired t-test, p=0.0209). **(E)** Time spent in the center zone of the open field, indicating no significant difference between groups (ctrl, n=8; EAE, n=10; 34.64 ± 5.547 vs 28.02 ± 9.644, Mann–Whitney U test, p=0.1728). **(F)** Pain sensitivity measured by latency to nociceptive response is decreased in EAE mice, indicating enhanced pain perception (ctrl, n=8; EAE, n=10; 14.96 ± 1.108 vs 10.93 ± 0.6615, unpaired t-test, p=0.0048). **(G)** Timeline of contextual fear-conditioning protocol. **(H)** Training (day 1): four 2 s, 1 mA shocks at 60-s intervals after a 3-min habituation; groups reached comparable freezing (ctrl, n=8; EAE, n=10). **(I)** Extinction (day 2, 20 min): EAE mice froze more than controls (ctrl, n=8; EAE, n=10; two-way ANOVA, group, F (1, 480) = 10.30, p = 0.0014). **(J)** Retrieval in context A (day 8, 4 min): EAE mice maintained higher freezing (ctrl, n=8; EAE, n=10; two-way ANOVA, group, F (1, 64) = 4.956, p = 0.0295). **(K)** Generalization test in context B (day 9, 4 min): markedly elevated freezing in EAE mice (ctrl, n=8; EAE, n=10; two-way ANOVA, group, F (1, 56) = 46.64, p < 0.0001). **(L)** Long-term retrieval in context A (day 15, 4 min): freezing remained higher in EAE mice (ctrl, n=8; EAE, n=10; two-way ANOVA, group, F (1, 64) = 10.12, p = 0.0023). Data are mean ± SEM; ctrl, control; EAE, experimental autoimmune encephalomyelitis. The symbols *, **, ***, and **** represent statistical significance at P < 0.05, P < 0.01, P < 0.001, and P < 0.0001, respectively.

EAE mice showed progressive weight loss beginning on day 14 post-immunization that paralleled the rise in neurological disability (**Figures 1A, B**). On day 18, in the open-field assay, travelled distance was reduced in the EAE mice (**Figures 1C, D**, unpaired t-test, p < 0.05), in agreement with previous studies demonstrating that locomotor distance is a sensitive early marker of motor dysfunction in EAE (18). The time in the center zone was unchanged, suggesting no obvious anxiety phenotype (**Figure 1E**). Finally, paw withdrawal latency on a 53 °C hotplate was significantly shortened in EAE mice (**Figure 1F**, unpaired t-test, p < 0.05), consistent with the thermal hyperalgesia described in neuroinflammatory demyelination models (19). Together, these data confirm that our mice developed the expected somatic and sensory hallmarks of EAE.

To evaluate the susceptibility of EAE mice to stress induced PTSD-like behaviors, we used a contextual fear-conditioning paradigm (**Figure 1G**). Contextual fear conditioning training began when animals reached a clinical score of 2 (see Method section for detailed description), about 20 days after EAE induction. After a 3-minute habituation, each mouse received four shocks (2 s long, 1 mA intensity, 60 s interval). EAE and control animals achieved comparable freezing levels during training (**Figure 1H**). Extinction was assessed in a 30−min session on day 2: EAE mice exhibited overall higher levels of freezing behavior compared to controls (**Figure 1I**, two-way ANOVA, main effect of group: F(1, 480) = 10.30, p = 0.0014). Re-exposure to context A on day 8 again revealed higher freezing in EAE mice across the 4-min test (**Figure 1J**, two-way ANOVA: main effect of group: F(1, 64) = 4.956, p = 0.0295), indicating that EAE mice retained more robust fear compared with controls. On day 9, mice were placed in a novel context B, a setting that differed entirely from context A, featuring a different floor texture and patterned walls. EAE mice exhibited markedly higher freezing than controls (**Figure 1K**, two-way ANOVA, main effect of group: F(1, 56) = 46.64, p < 0.0001), demonstrating pronounced fear generalization. On day 15, EAE mice still displayed significantly greater freezing in context A than controls (**Figure 1L**, two-way ANOVA: main effect of group, F (1, 64) = 10.12, p = 0.0023). Thus, when comparable initial acquisition was induced by intense foot shocks, EAE mice displayed impaired fear extinction and marked fear generalization.

### EAE-induced hippocampal glial activation and pyramidal layer atrophy

**Figure 2:**
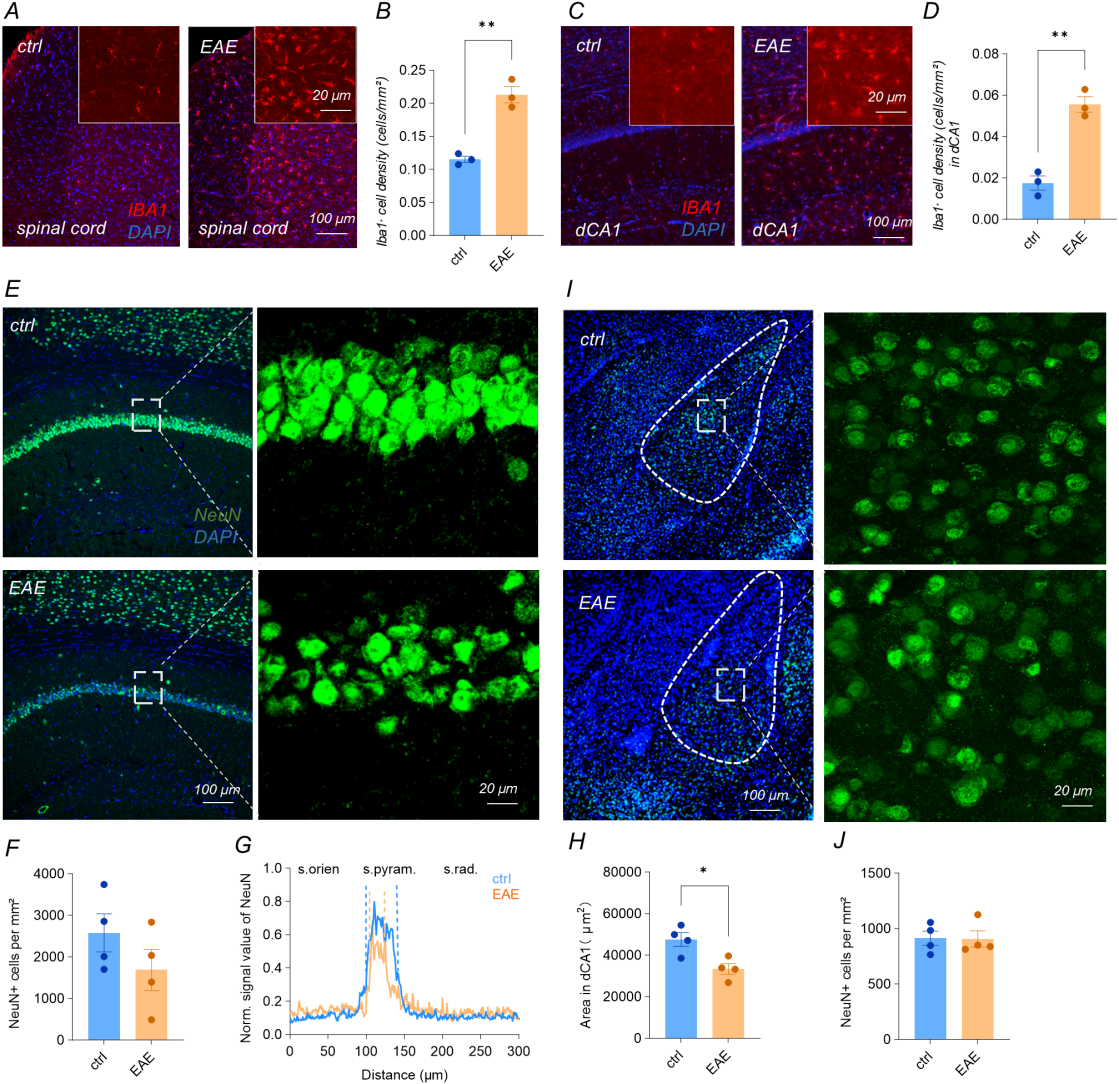
EAE-induced hippocampal glial activation and pyramidal layer atrophy. **(A-D)** IBA1 immunostaining revealed significantly increased amoeboid microglia in the lumbar spinal cord **(A, B)** (ctrl 27.92 ± 1.158 vs EAE 51.58 ± 3.008, unpaired t-test, p =0.0018; n = 3) and dorsal CA1 (dCA1) of EAE mice **(C, D)** (ctrl 3.778 ± 0.8678 vs EAE 11.67 ± 0.7698, unpaired t-test, p= 0.0024; n = 3). **(E–H)** NeuN staining in dCA1: neuronal density unaltered**(F)** (ctrl 2579 ± 458.4 vs EAE 1690 ± 496.5, unpaired t-test, p = 0.2364, n = 4), but pyramidal-layer full-width at half-maximum (FWHM) **(G)** (two-way ANOVA, F (471, 8496) = 21.04, P<0.0001; n = 4) and somatic cross-sectional area reduced **(H)** (ctrl 47496 ± 3355 vs EAE 33446 ± 2624, unpaired t-test, p =0.0164; n = 4) in EAE mice. **(I, J)** NeuN staining in the basolateral amygdala (BLA) showed no numerical or morphological changes **(J)** (ctrl 913.2 ± 65.29 vs EAE 906.9 ± 73.48, Mann–Whitney U test, p > 0.05; n = 4). Data are mean ± SEM. *: P<0.05, **: P<0.01.

Immunohistochemical staining for the microglial marker IBA1 showed a significant increase in IBA1-positive microglia in the lumbar spinal cord of EAE mice with a averaged clinical score of 2, where the cells also adopted the characteristic amoeboid morphology of activation (**Figures 2A, B**; unpaired t-test, n = 3 mice, p < 0.005). A comparable rise in microglial density and activation was observed in the dCA1 region of the hippocampus (**Figures 2C, D**; unpaired t-test, n = 3 mice, p < 0.005). Together, these findings demonstrate that EAE elicits widespread microglial activation extending from the spinal cord to the hippocampus, establishing a pro-inflammatory milieu throughout the neural circuitry.

To evaluate whether EAE-induced neuroinflammation extends beyond microglial activation to structural alterations in hippocampal neurons, we examined NeuN immunoreactivity in mice with a clinical score of 2. In the dCA1, a region crucial for contextual fear memory (**Figure 2E**), the density of NeuN−positive neuron density of EAE mice was similar to that from control mice (**Figure 2F**, p > 0.05, unpaired t-test, n = 4 mice). However, the full−width at half−maximum of the NeuN fluorescence profile, an index of pyramidal−layer thickness, was markedly narrower in EAE mice and their mean somatic cross−sectional area was significantly reduced (**Figures 2G, H**, P < 0.05, unpaired t-test, n = 4 mice). These findings indicate somatic shrinkage rather than overt neuronal loss.

By contrast, neurons in the basolateral amygdala (BLA), another node of the fear circuitry, showed no significant morphological or numerical alterations (**Figures 2I, J**, unpaired t-test, n = 4 mice).

Collectively, EAE provokes widespread microglial activation and selectively thins the hippocampal CA1 pyramidal layer via neuronal soma shrinkage, while leaving basolateral amygdala neurons morphologically intact, highlighting a hippocampus-biased structural vulnerability within the fear circuit.

### Reduced c-Fos expression in hippocampal pyramidal layer cells in EAE model

The medial prefrontal cortex (mPFC), basolateral amygdala (BLA), and dorsal CA1 (dCA1) form an integrated circuit that mediates the encoding, retrieval, and extinction of contextual fear. To determine whether EAE alters basal neuronal activation within this limbic circuitry, we quantified c-Fos protein expression (an immediate-early gene product) in the mPFC, BLA, and dCA1 of mice exhibiting an averaged EAE clinical score of 2.

**Figure 3:**
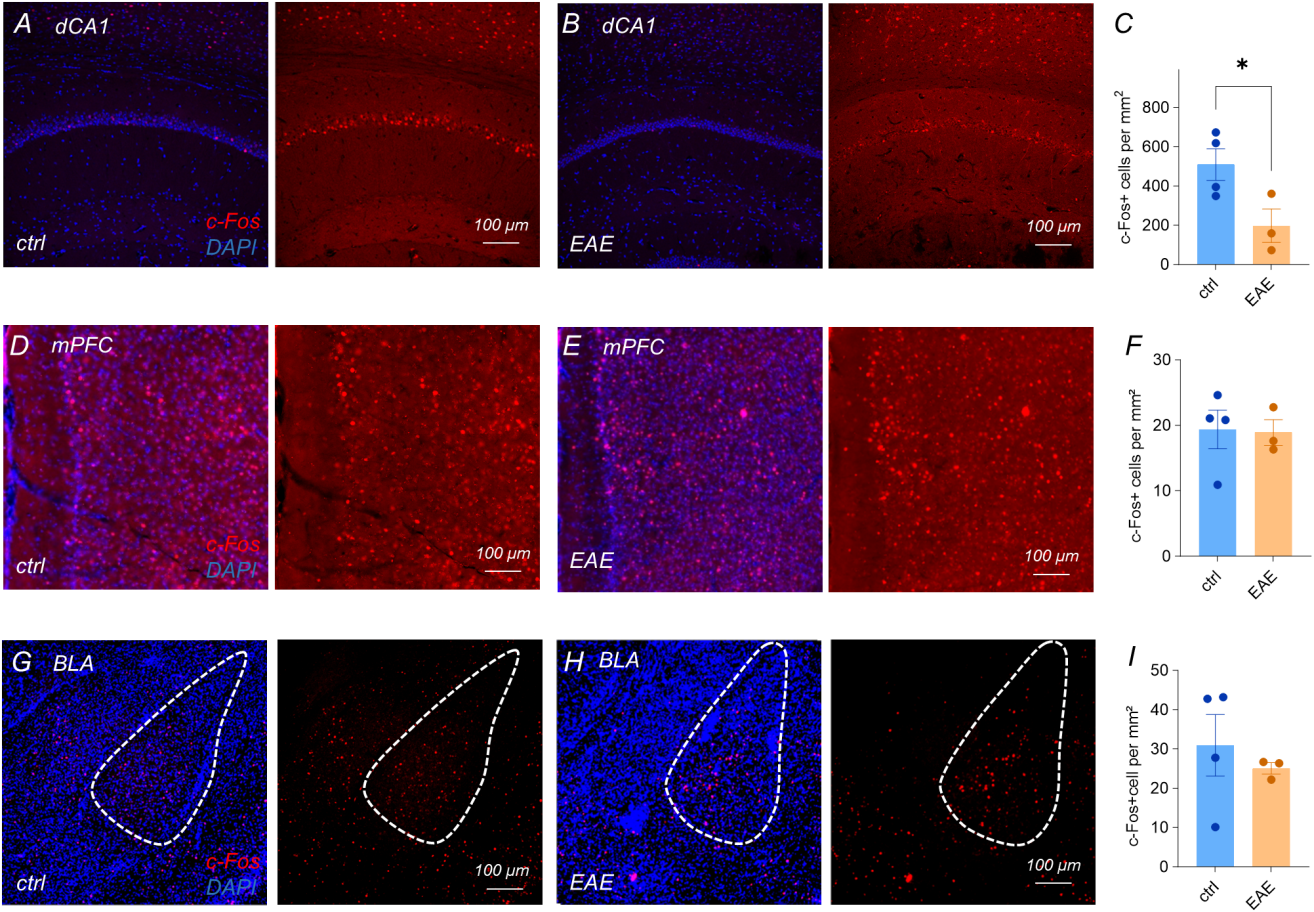
Reduced c-Fos expression in hippocampal pyramidal layer cells in EAE model. **(A-C)** c-Fos-positive nuclei in dCA1 pyramidal layer decreased in EAE mice (ctrl 508.7 ± 80.18 vs EAE 197.8 ± 85.17, unpaired t-test, p =0.0471; ctrl n = 4, EAE n = 3). **(D-F)** mPFC (ctrl 19.38 ± 2.944 vs EAE 18.91 ± 1.968, unpaired t-test, p > 0.05, ctrl n = 4, EAE n = 3) and **(G-I)** BLA show no group differences (ctrl 30.97 ± 7.808 vs EAE 25.08 ± 1.429, unpaired t-test, p > 0.05, ctrl n = 4, EAE n = 3). Data represent mean ± SEM. *: P<0.05.

EAE mice showed a significant reduction in c-Fos immunoreactivity within the pyramidal layer of dCA1 (**Figures 3A–C**; unpaired t-test, ctrl n = 4, EAE n = 3, p < 0.05). By contrast, confocal analyses revealed no group differences in the density of c-Fos-positive nuclei in either the mPFC (**Figures 3D–F**; unpaired t-test, p > 0.05) or the BLA (**Figures 3G–I**; unpaired t-test, p > 0.05).

These findings indicate that EAE selectively dampens activity-dependent gene expression in hippocampal pyramidal neurons, while sparing prefrontal and amygdala excitatory populations.

### EAE induces hypoexcitability in dorsal CA1 pyramidal neurons

**Figure 4:**
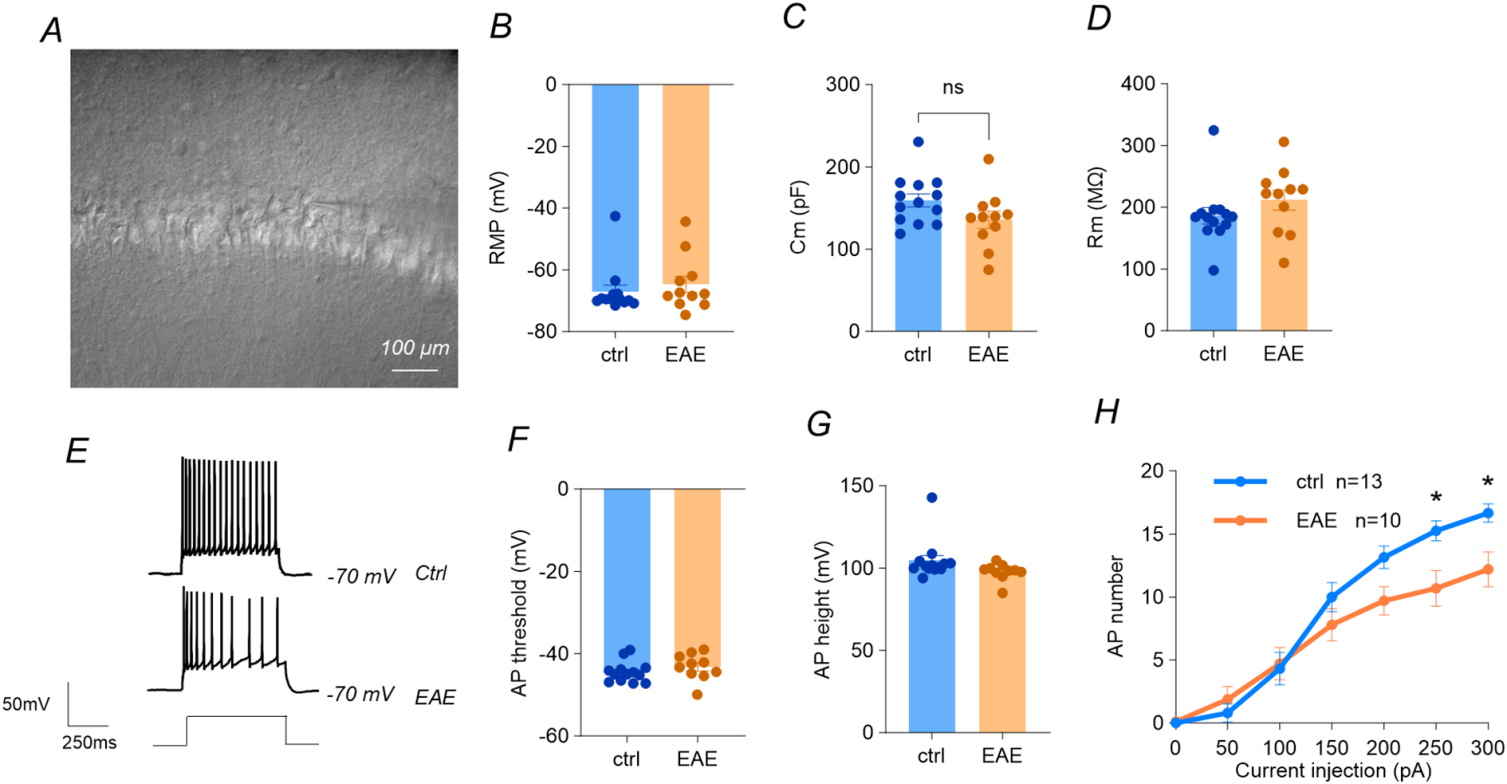
EAE induces hypoexcitability in dorsal hippocampal pyramidal neurons. **(A)** Whole-cell current-clamp configuration in hippocampal slices. **(B–D)** Resting membrane potential (RMP), capacitance (CM) and input resistance (RM) unchanged (unpaired t-tests, p> 0.05, ctrl n = 13, EAE n= 11, cells from 3 mice per group). **(E-G)** AP threshold slope and amplitude unaffected (unpaired t-tests, p> 0.05, ctrl n = 13, EAE n= 11, cells from 3 mice per group). **(H)** Frequency-current relation: EAE neurons fire fewer spikes across 50–300 pA steps (two-way ANOVA, group, F (1, 139) = 11.86, p = 0.0008, cells from 3 mice per group). Data represent mean ± SEM. *: P<0.05.

To explore the neuronal mechanism underlying the reduced c-Fos staining in dCA1 of EAE mice, we performed whole−cell current−clamp recordings from dorsal CA1 pyramidal neurons (**Figure 4A**). Passive membrane properties did not differ between EAE and control groups (P > 0.05) including resting membrane potential (RMP; **Figure 4B**, membrane capacitance (Cm; **Figure 4C**), and input resistance (Rm; **Figure 4D**). Interestingly, although the capacitance between two groups did not differ, the capacitance from EAE mice indeed showed a smaller trend compared to control mice (**Figure 4C**, 135.8 ± 10.47 vs 159.3 ± 8.2 pF, p = 0.087, unpaired t test, for EAE vs control, n = 11 and 13 respectively), indicating a smaller membrane size of dCA1 pyramidal neurons in agreement with the shrinkage of pyramidal layer in EAE mice as shown in **Figures 2E–H**. Further, neither the slope of the action−potential (AP) threshold nor AP amplitude was altered by EAE (**Figures 4E-G**), indicating that disease does not influence excitability by modifying passive properties or AP threshold.

In contrast, the frequency-current curve revealed a clear loss of firing gain in EAE neurons (**Figure 4H**). During 600-ms depolarizing steps delivered in 50 pA increments, EAE neurons fired fewer spikes than controls (Two-way ANOVA, group: F_(1, 139)_ = 11.86, p=0.0008). Thus, across the entire current range, EAE pyramidal neurons generated fewer action potentials than controls, indicating a pronounced reduction in intrinsic excitability.

Collectively, these findings demonstrate decreased intrinsic excitability of CA1 pyramidal neurons in EAE, which may underly the reduced c-Fos staining phenotypes of EAE mice (all recordings obtained from individual cells of three mice per group).

### Chemogenetic re-activation of dCA1 pyramidal neurons mitigates the fear-extinction deficit in EAE mice

**Figure 5:**
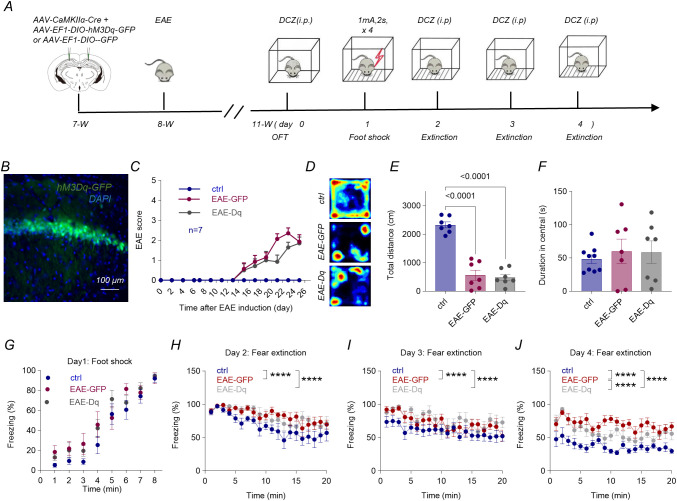
Chemogenetic activation of dCA1 pyramidal neurons rescues late-stage extinction in EAE mice. **(A)** Protocol: AAV-CaMKIIα-Cre plus AAV-EF1α-DIO-hM3Dq-GFP(Dq) or AAV-EF1α-DIO-GFP injected into 7-week-old female C57BL/6 mice. EAE induction one-week post-surgery; deschloroclozapine (DCZ, 0.3 mg kg^-^¹ i.p.) given 1 h before open-field (OFT) and each fear session. **(B)** hM3Dq-GFP expression confined to dCA1 pyramidal neurons. **(C)** Clinical EAE scores (0–5). **(D-F)** OFT (3 weeks post-immunization): Total locomotor distance was significantly reduced in both EAE groups (EAE-GPP and EAE-Dq) compared to controls (P<0.0001 for ctrl vs. EAE-GFP; p<0.0001 for ctrl vs. EAE-Dq, one-way ANOVA, n = 7, p < 0.001); center-time unchanged (P = 0.8134 for ctrl vs. EAE-GFP; p=0.8433 for ctrl vs. EAE-Dq, one-way ANOVA, n = 7, p> 0.05). **(G)** Conditioning protocol (four 2s, 1mA shocks). **(H)** Extinction (day 2): both EAE groups (EAE-GFP and EAE-Dq) exhibited significantly impaired fear extinction compared to controls over 20 min, with no significant difference between EAE-GFP and EAE-Dq groups (ctrl: 62.77 ± 2.06, EAE-GFP: 83.51 ± 2.06, EAE-Dq: 82.45 ± 2.06. Statistical analysis was performed using two-way ANOVA followed by Tukey’s multiple comparisons test. Tukey’s *post hoc* test revealed significant differences between ctrl and EAE-GFP (****p < 0.0001) and between ctrl and EAE-Dq (****p < 0.0001), but not between EAE-GFP and EAE-Dq (ns, p = 0.9014).). **(I)** Retrieval-1 (day 3): both EAE groups (EAE-GFP and EAE-Dq) exhibited similar freezing behavior during 20 min fear extinction training (ctrl: 57.08 ± 2.68, EAE-GFP: 71.94 ± 2.68, EAE-Dq-GFP: 76.93 ± 2.68. Statistical analysis was performed using two-way ANOVA followed by Tukey’s multiple comparisons test. Tukey’s *post hoc* test revealed significant differences between ctrl and EAE-GFP (****p < 0.0001) and between ctrl and EAE-Dq-GFP (****p < 0.0001), but not between EAE-GFP and EAE-Dq-GFP (ns, p = 0.1518). n = 7). **(J)** Retrieval-2 (day 4): EAE-GFP mice showed significantly higher freezing levels than both control and EAE-Dq mice during the extinction session on day 4 (ctrl:36.12 ± 1.62, EAE-GFP: 70.41 ± 1.62, EAE-Dq-GFP: 58.36 ± 1.62. Statistical analysis was performed using two-way ANOVA followed by Tukey’s multiple comparisons test. Tukey’s *post hoc* test revealed significant differences between ctrl and EAE-GFP (****p < 0.0001), between ctrl and EAE-Dq-GFP (****p < 0.0001), and between EAE-GFP and EAE-Dq-GFP (****p < 0.0001).), indicating impaired extinction. In contrast, EAE-Dq mice exhibited reduced freezing, comparable to control levels, suggesting that dCA1 activation mitigated the extinction deficit. Data are mean ± SEM.

Next, we first stereotaxically infused a virus cocktail (AAV-CaMKIIα-Cre together with either AAV-DIO-hM3Dq-GFP or AAV-DIO-GFP) into the dorsal CA1 (dCA1) of 7-week-old female C57BL/6 J mice (**Figure 5A**). One week later, experimental autoimmune encephalomyelitis (EAE) was induced. Immunofluorescence confirmed that hM3Dq expression was restricted to dCA1 pyramidal neurons (**Figure 5B**).

EAE clinical signs emerged ~12 days post-induction and plateaued at an average score of 2 by day 20 (defined as day 0 of behavioral testing; **Figures 5C**, n = 7 per group). Beginning on day 0, mice received the DREADD (Designer Receptors Exclusively Activated by Designer Drugs)agonist deschloroclozapine (DCZ; 0.3 mg/kg, i.p.) 60 min before each behavioral session to selectively activate hM3Dq-DREADD–expressing neurons. The chemogenetic manipulation (hM3Dq-DREADD activation by DCZ) was performed consistently throughout all behavioral procedures.Compared with control mice, both EAE-GFP and EAE-Dq animals exhibited a marked reduction in total distance travelled (**Figures 5D, E**; one-way ANOVA), whereas time spent in the center zone was unchanged across groups (**Figure 5F**; p > 0.05). Thus, EAE reduced spontaneous locomotion without altering baseline anxiety, and acute activation of dCA1 excitatory neurons did not influence either variable.

To determine whether chemogenetic activation of dCA1 excitatory neurons could modulate fear extinction in EAE mice, we employed a contextual fear‐conditioning paradigm. All groups showed comparable increases in freezing across the four 1 mA foot shocks, indicating intact fear acquisition (**Figures 5G**, Two-way ANOVA, p > 0.05).

During the 20-min extinction sessions, freezing declined across days in all groups but remained significantly higher in EAE-GFP and EAE-Dq mice than in controls throughout the training period (**Figures 5H–J**). As expected, repeated re-exposure to the same context led to a gradual reduction of conditioned fear. Notably, the difference between the two EAE groups emerged only by day 4, likely reflecting the cumulative nature of fear extinction: early sessions were dominated by a strong residual fear memory, whereas continued fear extinction progressively unmasked the beneficial effect of enhancing dCA1 excitability. By day 4, EAE-Dq mice froze significantly less than EAE-GFP mice, indicating improved extinction performance.

To assess whether chemogenetic stimulation effectively enhanced neuronal activation in dorsal CA1 under EAE conditions, mice were perfused 2 h after DCZ administration and brain tissue was collected for analysis. Quantification of c-Fos expression in GFP-labeled pyramidal neurons revealed that EAE-Gq mice exhibited a significantly higher proportion of c-Fos^+^/GFP^+^ cells compared with EAE-GFP controls, indicating that hM3Dq activation robustly increased excitatory neuron activity(**Supplementary Figure 1**).

Collectively, these data demonstrate that hypo-excitability of dorsal hippocampal pyramidal neurons is a key contributor of the impaired fear-extinction learning that underlies PTSD-like behavior in EAE mice. Restoring dCA1 activity via chemogenetic stimulation selectively rescued the extinction deficit without alleviating motor impairment or altering anxiety, highlighting dorsal CA1 as a promising therapeutic node for comorbid stress disorders in demyelinating disease.

### EAE reprogram gene networks that sustain intrinsic excitability of CA1 projection neurons

**Figure 6:**
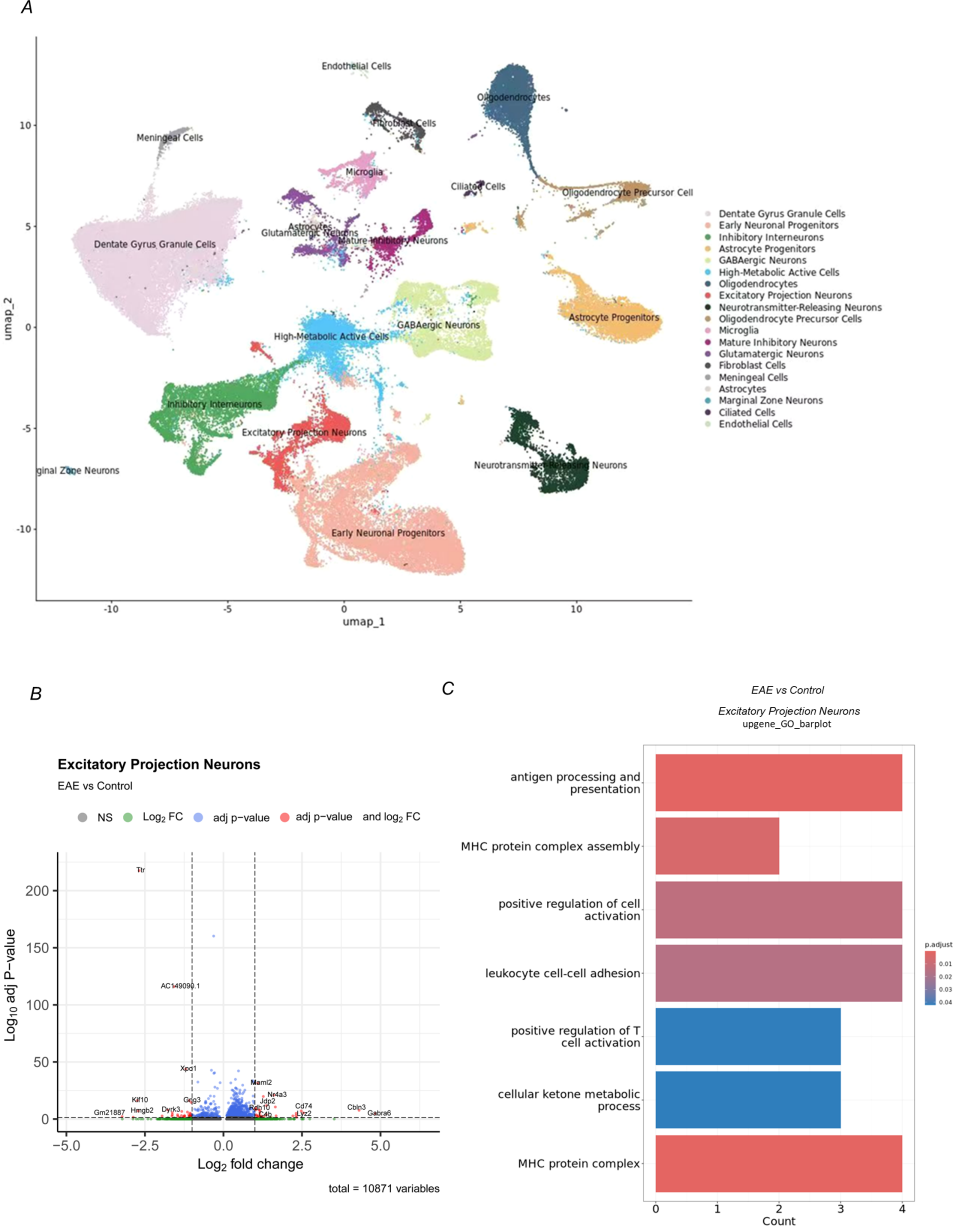
Single-nucleus RNA sequencing reveals excitatory neuron heterogeneity in EAE hippocampus. **(A)** UMAP embedding of 19 cell clusters from control and EAE hippocampus. **(B)** Volcano plot of all detected genes in CA1 excitatory neurons (n = 10, 871). Genes with FDR < 0.05 are highlighted; Up32 and Down32 denote robust DE genes defined as FDR < 0.05 and log_2_FC > 1 (Up32) or log_2_FC < −1 (Down32). **(C)** GO enrichment analysis of upregulated genes in excitatory projection neurons. (n = 3 mice per group).

So far, we have demonstrated that EAE mice exhibit persistent context-generalized fear, a core behavioral phenotype of post-traumatic stress disorder (PTSD). This pathological behavior is accompanied by marked somatic atrophy of hippocampal CA1 pyramidal neurons, microglial hyperactivation, and a significant reduction in neuronal firing gain, indicating that dorsal hippocampal excitatory neurons undergo both structural and functional impairments in EAE. To uncover the molecular basis of these abnormalities, we performed single-nucleus RNA sequencing (snRNA-seq) on hippocampal tissue from EAE mice (clinical score ≈ 2, n = 3 per group) and age-matched controls. After stringent quality control and batch correction, UMAP-based dimensionality reduction identified 19 distinct cell populations, including excitatory and inhibitory neurons, glial cells, and vascular-associated cell types (**Figure 6A**). Within the neuronal clusters, we further annotated major subtypes such as excitatory projection neurons, inhibitory interneurons, GABAergic neurons, and neurotransmitter-releasing neurons, thereby establishing a refined cellular framework for subtype-specific transcriptomic analyses.

Focusing on CA1 excitatory projection neurons, we examined all 10, 871 detected genes(**Supplementary Table 1**), each corresponding to one dot in **Figure 6B**. Using an adjusted p-value cutoff (adj p < 0.05), 1, 162 genes were identified as significantly differentially expressed (red + blue dots in **Figure 6B**). Among these, applying a more stringent fold-change threshold revealed 32 strongly upregulated genes (log_2_FC > 1; “Up32”) and 32 strongly downregulated genes (log_2_FC < −1; “Down32”) (**Figure 6B**; **Supplementary Table 2**).

GO enrichment analysis was performed on the Up32 and Down32 gene sets, with significance defined as adjusted p-value < 0.05 (**Figure 6C**; **Supplementary Table 3**). The Up32 genes showed significant enrichment for immune- and cytokine-associated programs, including antigen processing and presentation, MHC protein complex–related pathways, and leukocyte/T-cell activation–related processes (**Figure 6C**). In contrast, no GO terms for the Down32 set remained significant after multiple-testing correction (adjusted p-value < 0.05).

Together, snRNA-seq reveals a focused reprogramming of CA1 excitatory projection neurons in EAE, with a discrete set of strongly regulated genes (Up32/Down32). The Up32 signature is enriched for immune/cytokine pathways, consistent with an inflammation-linked neuronal state. This molecular shift provides a plausible transcriptomic correlate of the CA1 structural atrophy and reduced firing gain observed in EAE, linking neuroinflammatory remodeling to impaired intrinsic excitability and fear generalization.

## Discussion

In this study, we show that EAE mice exhibit persistently impaired fear extinction together with marked generalization of contextual fear, indicating substantial disruption of hippocampal-dependent fear regulation. We identify pronounced hypoexcitability and structural alterations of dorsal CA1 pyramidal neurons as key contributors to this behavioral phenotype, and demonstrate that chemogenetic activation of these neurons partially restores extinction performance. Furthermore, single-nucleus RNA sequencing reveals a distinct transcriptional reprogramming in CA1 excitatory neurons, characterized by activation of neuroimmune gene programs. Together, these findings suggest that dorsal CA1 excitatory neurons undergo coordinated structural, functional, and transcriptional alterations during EAE, changes that may participate in the processes leading to impaired fear-memory extinction under neuroinflammatory conditions.

### Dorsal CA1 hypoexcitability as a potential substrate for impaired fear extinction in EAE

The hippocampal CA1 subregion plays a pivotal role in contextual fear memory formation and extinction (20). Consistent with previous reports demonstrating impaired CA1 long-term potentiation (LTP) in EAE mice (21). In our EAE model, reduced c-Fos expression and decreased pyramidal neuron excitability were observed in CA1. Chemogenetic activation of neuronal excitability partially rescued extinction deficits, indicating that hypoexcitability of dorsal hippocampal pyramidal neurons constitutes a key mechanism underlying impaired fear extinction in EAE. A comparable volumetric reduction is a well-replicated biomarker of PTSD and a prospective risk factor for its development (22). Together, these human and pre-clinical data support the idea that hippocampal integrity is a convergent vulnerability node for MS and PTSD.

### Transcriptomic reprogramming of hippocampal pyramidal neurons during EAE pathogenesis

snRNA-seq of hippocampal excitatory neurons during EAE pathogenesis revealed a prominent neuroimmune-like transcriptional response in CA1 excitatory neurons. Upregulated genes were strongly enriched for immune- and interferon-associated pathways, including antigen processing and presentation, MHC protein complex assembly, and leukocyte/T-cell activation–related programs, together with apoptosis-related terms such as leukocyte apoptotic process and cysteine-type endopeptidase activator activity involved in apoptotic process. This neuron-associated immune signature is consistent with robust activation of microglia and astrocytes in EAE and multiple sclerosis, where glial cells amplify neuroinflammatory cascades within the hippocampal microenvironment (23). Notably, cytokines and reactive oxygen/nitrogen species released in inflammatory milieus can remodel neuronal membrane properties and modulate ion-channel gating, providing a mechanistic route through which immune signaling may translate into altered intrinsic excitability (24–27).

Beyond serving as passive targets, neurons can upregulate antigen-processing and MHC-related programs under inflammatory conditions (28, 30). Neuronal MHC signaling has been implicated in synaptic remodeling and plasticity, and its aberrant induction may heighten vulnerability to immune-mediated interactions, thereby exacerbating local circuit dysfunction (29–31). Together, these findings support the view that immune-program activation in CA1 excitatory neurons reflects a neuron-intrinsic inflammatory state that plausibly links neuroinflammation to impaired hippocampal excitability and extinction-related behavioral deficits.

### Therapeutic implications

The immune-like transcriptional activation observed in hippocampal pyramidal neurons suggests that neurons may actively contribute to neuroinflammatory disease processes rather than acting solely as passive recipients of glial-driven inflammation. Neuronal expression of immune-related molecules, including components of antigen presentation, has been documented across developmental and pathological contexts (28–30). This raises the possibility that maladaptive engagement of these programs in EAE could participate in circuit instability and biased threat processing. Importantly, the partial reversal of extinction deficits by chemogenetic activation highlights dorsal CA1 as a tractable node for neuromodulatory intervention. Future studies should determine whether pharmacological or optogenetic strategies can produce durable rescue across disease stages and whether such approaches can mitigate comorbid PTSD-like symptoms in neuroinflammatory disorders such as MS.

### Limitations and future directions

This study profiled a single disease stage (clinical score ≈ 2). Longitudinal sequencing and physiology will be required to map the temporal sequence from immune activation to hypo−excitability. In addition, sex−specific effects were not explored and warrant investigation given known differences in MS prevalence and PTSD vulnerability.

In sum, our work delineates a convergent immune–excitability mechanism that compromises dorsal hippocampal CA1 function and sustains persistent fear in EAE. By integrating electrophysiology, single-cell genomics and circuit-specific rescue, we provide a coherent framework linking neuro-immune signaling to behavioral pathology and identify candidate targets for therapeutic intervention.

## Data Availability

The raw sequence data reported in this paper have been deposited in the Genome Sequence Archive (Genomics, Proteomics & Bioinformatics 2021) in National Genomics Data Center (Nucleic Acids Res 2022), China National Center for Bioinformation / Beijing Institute of Genomics, Chinese Academy of Sciences (GSA: CRA029963) that are publicly accessible at https://ngdc.cncb.ac.cn/gsa.

## Supplementary material

Supplementary Figure 1 Chemogenetic activation increases c-Fos expression in dorsal CA1 pyramidal neurons of EAE mice.**(A, B)** Representative confocal images showing DAPI, c-Fos, and GFP signals in dorsal CA1 of EAE-GFP and EAE-Gq mice following DCZ administration. Compared with EAE-GFP mice, EAE-Gq animals displayed a higher proportion of c-Fos^+^/GFP^+^ pyramidal neurons, as quantified on the right (mean ± SEM; n=4, p < 0.05). Scale bars, 100 μm.
