# Supplementary figures and images for: Hippocampal neuronal hypoexcitability contributes to PTSD-like phenotypes in the experimental autoimmune encephalomyelitis model
Source: Front Psychiatry. 2026 May 20;17:1683599. doi: 10.3389/fpsyt.2026.1683599 (PMC13231496; doi:10.3389/fpsyt.2026.1683599)

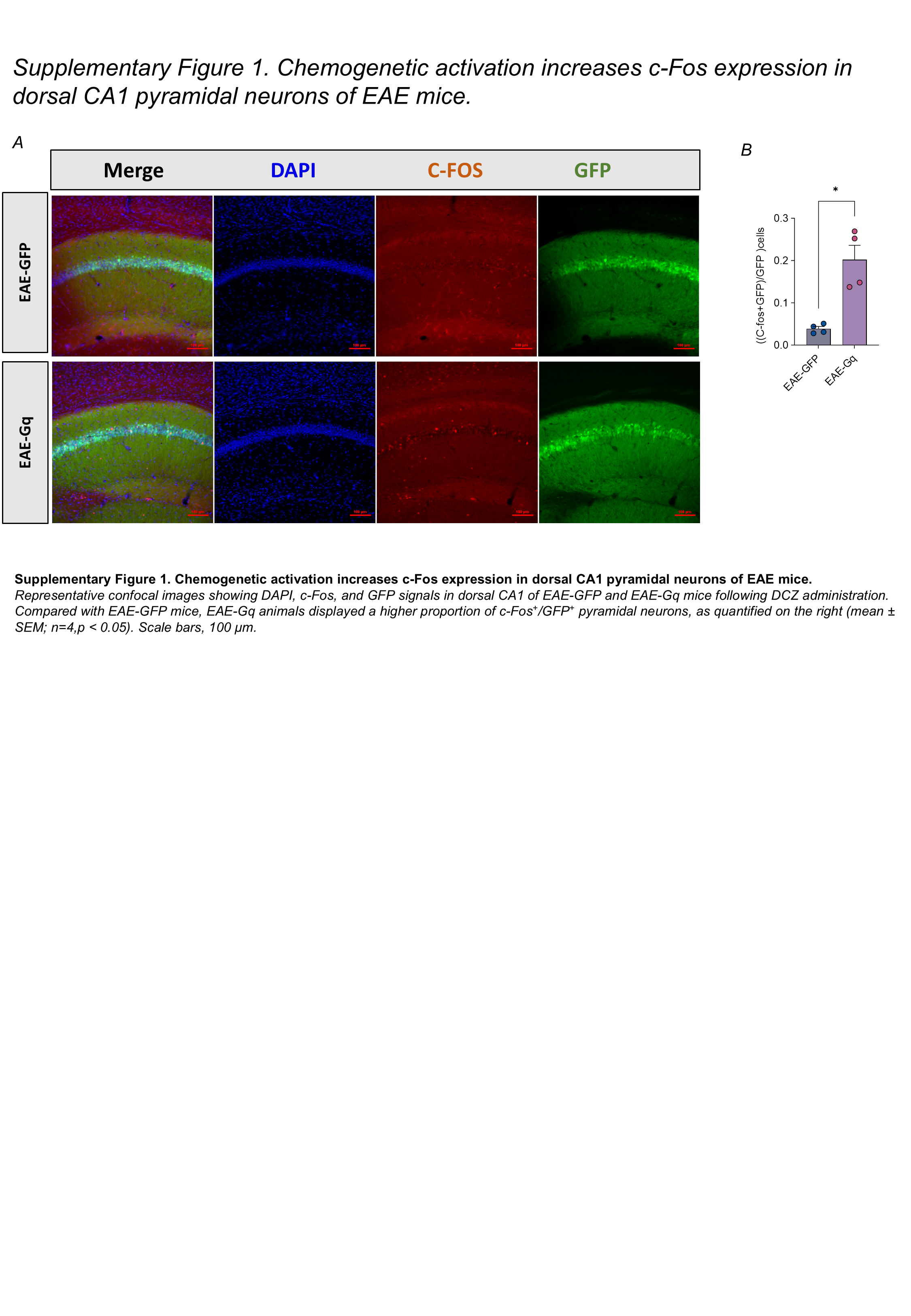

Supplement: Supplementary Figure 1 — Chemogenetic activation increases c-Fos expression in dorsal CA1 pyramidal neurons of EAE mice.(A, B) Representative confocal images showing DAPI, c-Fos, and GFP signals in dorsal CA1 of EAE-GFP and EAE-Gq mice following DCZ administration. Compared with EAE-GFP mice, EAE-Gq animals displayed a higher proportion of c-Fos+/GFP+ pyramidal neurons, as quantified on the right (mean ± SEM; n=4, p < 0.05). Scale bars, 100 μm. [file Image1.tif]
